# Variants of unknown significance in fetal heart malformations: distribution and impact on prenatal decision-making

**DOI:** 10.3389/fped.2025.1605899

**Published:** 2025-09-24

**Authors:** Qingsong Wang, Jun Yin, Xiaomeng Zhang, Huimin Ou, Fuyan Li, Yundong Zhang, Caiyu Guo, Weiyi Wan, Yongyu Cao, Tongyong Luo, Xianmin Wang

**Affiliations:** ^1^ Department of Pediatrics, West China Hospital Sichuan University Jintang Hospital, Jintang First People’s Hospital, Chengdu, Sichuan, China; ^2^ Department of Ultrasound, West China Hospital Sichuan University Jintang Hospital, Jintang First People’s Hospital, Chengdu, Sichuan, China; ^3^ Pediatric Cardiology Center, Sichuan Provincial Women’s and Children’s Hospital/The Affiliated Women’s and Children’s Hospital of Chengdu Medical College, Chengdu, Sichuan, China

**Keywords:** fetal heart malformations, variants of unknown significance (VUS), prenatal diagnosis, genetic counseling, chromosomal microarray analysis (CMA)

## Abstract

**Objective:**

To investigate the distribution patterns of variants of unknown significance (VUS) in fetuses with heart malformations (CHD) combined with extracardiac abnormalities and their impact on prenatal decision-making.

**Methods:**

A retrospective analysis was conducted on the chromosomal microarray analysis (CMA) data of 697 cases of fetal heart malformations (including simple, complex, and combined with extracardiac abnormalities) and 2,689 controls from Sichuan Provincial Maternal and Child Health Care Hospital between January 2020 and August 2022. Copy number variants (CNVs) were classified according to the ACMG guidelines (pathogenic, VUS, benign), and the differences in VUS detection rates and their impact on pregnancy outcomes were compared among groups.

**Results:**

Among 697 fetuses with prenatally diagnosed cardiac malformations, 602 (86.37%) had simple, 69 (9.90%) complex, 18 (2.58%) combined with structural extracardiac anomalies, and 8 (1.15%) with soft markers. Karyotype abnormalities occurred in 4.74% (26/549), 16.36% (9/55), 27.78% (5/18), and 12.50% (1/8) of these groups, respectively, all exceeding controls (4.71%, *P* < 0.05). Pathogenic CNVs were detected in 4.88% (27/553), 7.69% (5/65), 8.33% (2/24), and 0% (0/2), respectively; the first three rates were significantly higher than controls (1.38%, *P* < 0.05, *P* = 0.002, *P* = 0.033). VUS rates rose progressively: 0.54% (3/553), 1.54% (1/65), 12.50% (3/24), and 100% (2/2). Among nine VUS-positive pregnancies, six resulted in live-born infants without abnormalities; three were terminated due to additional malformations or parental anxiety.

**Conclusion:**

Fetuses with cardiac malformations accompanied by structural extracardiac anomalies carry the highest genetic risk; karyotyping combined with CMA should therefore be performed routinely. Complex cardiac malformations also warrant concurrent testing, whereas simple malformations and those with soft markers can be evaluated individually.

## 1 Introduction

Heart malformations (heart malformations) is one of the most common birth defects in neonates, with an incidence rate of approximately 0.8%–1.2% globally (1). Heart malformations encompasses developmental malformations of the heart, aorta, or other major blood vessels, ranging from isolated anomalies (such as atrial or ventricular septal defects, isolated valvular dysplasia) to complex defects arising from primary developmental errors (such as tetralogy of Fallot, hypoplastic left heart syndrome). Despite significant reductions in neonatal mortality from heart malformations due to advanced medical interventions, heart malformations remains an important cause of stillbirth, neonatal disability, or death (2). Additionally, approximately 13% of heart malformations patients have defects in structures or functions outside the heart, Neurodevelopmental disorders may co-occur with heart malformations as part of syndromic conditions.

The etiology of heart malformations is complex, involving the interplay of genetic and environmental factors (3). Genetic factors play a significant role in the pathogenesis of heart malformations, including monogenic diseases, chromosomal abnormalities, and polygenic diseases (4). Chromosomal abnormalities are present in about 20% of heart malformations patients, with numerical chromosomal abnormalities being the most common, accounting for approximately 86%, mainly manifested as monosomy, aneuploidy, and polyploidy [based on observations from the second trimester (15–20 weeks) of pregnancy] (5). In recent years, with the development of genomics technology, an increasing number of gene variants associated with heart malformations have been discovered. These variants may lead to chromosomal structural variations, thereby affecting the pathogenesis of heart malformations.

Genetic diagnosis aids in the early diagnosis of heart malformations, guiding further assessment of heart malformations combined with other extracardiac abnormalities, providing prognostic information, facilitating genetic counseling, and defining reproductive recurrence risks. Chromosomal microarray analysis (CMA) is a high-resolution molecular genetic testing method that can detect small copy number variants (CNVs) in the genome, including microdeletions and microduplications (6). CMA technology overcomes the limitation of low resolution in traditional karyotype analysis and can detect CNVs as small as 50 kb (7). The application of CMA technology in prenatal diagnosis has become increasingly widespread, especially in the genetic etiology screening of fetal heart malformations, where it has become an important diagnostic tool (8). However, clinically significant CNVs of unknown significance (VUS) often appear in CMA test results. VUS refers to CNVs detected in the genome whose pathogenicity has not been established and whose association with disease phenotypes cannot be determined. Although CMA can detect CNVs as small as 50 kb, it cannot identify balanced structural rearrangements or single nucleotide variants. The presence of VUS poses significant challenges to clinical decision-making and genetic counseling (9).

In this study, we focus on the distribution characteristics of VUS in different types of heart malformations (simple, complex, combined with extracardiac abnormalities) and their impact on clinical decision-making. Although the overall detection rate of VUS is not high, it is significantly higher in heart malformations combined with structural extracardiac malformations than in other types, suggesting that this type of heart malformations may involve a more complex genetic background. By analyzing the distribution characteristics of VUS in depth, we hope to provide more accurate genetic counseling suggestions for clinicians and references for future research and clinical practice.

## 2 Materials and methods

### 2.1 Study subjects

This study employed a retrospective analysis, collecting data from 3,386 pregnant women who underwent amniocentesis at the Sichuan Provincial Maternal and Child Health Care Hospital between January 2020 and August 2022. Among them, 697 pregnant women whose fetuses were diagnosed with cardiac abnormalities (with or without extracardiac abnormalities) by fetal echocardiography were included in the experimental group. The inclusion criteria for the experimental group were: (1) Pregnant women diagnosed with fetal cardiac abnormalities (with or without extracardiac abnormalities) by two physicians with relevant qualifications; (2) Pregnant women who agreed to invasive prenatal diagnosis after prenatal genetic counseling and signed the informed consent form. The exclusion criteria were: (1) Pregnant women with contraindications to invasive prenatal diagnosis, or those who refused invasive prenatal diagnosis after prenatal consultation; (2) Fetal cardiac phenotypes involving only arrhythmia or pericardial effusion. The control group consisted of 2,689 cases who underwent karyotype analysis and/or copy number variation analysis after amniocentesis, but whose fetuses showed no developmental abnormalities on ultrasound [such as advanced maternal age (≥35 years), adverse obstetric history, non-invasive DNA indicating high risk of trisomy 21, family history of genetic diseases, etc.].

The experimental group was divided into four groups according to the birth defect diagnosis standards stipulated by the National Health Commission of China: (1) Simple heart malformations, a total of 602 cases: Refers to single cardiac lesions that generally do not cause hemodynamic changes, including isolated ventricular septal defects, mild aortic valve stenosis, and mild pulmonary valve stenosis; (2) Complex heart malformations, a total of 69 cases: Refers to the simultaneous existence of one or more cardiac and major blood vessel structural malformations, accompanied by significant hemodynamic changes, mainly including tetralogy of Fallot, transposition of the great arteries, single ventricle, double outlet right ventricle, complete atrioventricular septal defect, truncus arteriosus, pulmonary atresia, total anomalous pulmonary venous return, hypoplastic left heart syndrome, aortic arch interruption, coarctation of the aorta, and heterotaxy syndrome; (3) Heart malformations combined with structural extracardiac malformations, a total of 18 cases: Including malformations in other systems outside the circulatory system, such as esophageal atresia, intestinal dilation, renal pelvis separation, and clubfoot; (4) Heart malformations combined with soft markers, a total of 8 cases: Including soft markers such as absent nasal bone, increased fetal nuchal translucency, and choroid plexus cysts. Cases involving one or more types of complex heart malformations were counted as one case of complex heart malformations, and multiple simple heart malformations existing simultaneously were not classified as complex heart malformations.

Of the 697 fetal heart malformations cases initially enrolled, 644 underwent complete CMA analysis. The remaining 53 cases were excluded from CMA testing for the following reasons: 45 cases (6.5% of total) had insufficient DNA yield (<10 ng/μl as measured by Qubit fluorometry) from amniotic fluid samples despite using the QIAamp DNA Mini Kit extraction protocol, while 8 cases (1.1%) were excluded due to parental refusal of additional genetic testing after normal karyotyping results. Comparative analysis showed no significant differences in baseline characteristics (including heart malformations classification and maternal age) between included and excluded cases (all *p*-values > 0.05), suggesting minimal impact of these exclusions on the study's overall findings.

### 2.2 Ultrasonography

A GE Voluson E8 color Doppler ultrasound diagnostic instrument (equipped with a 4–8 MHz probe and an abdominal volume probe) was utilized, following a standardized protocol: initially, fetal position and cardiac activity were confirmed. Systematic fetal cardiac evaluation was performed through multiple planes, including four-chamber views, left and right ventricular outflow tract sections, vascular sections, long-axis sections of the inferior vena cava, short-axis sections of the great arteries, aortic arch sections, and arterial duct arch sections, to assess atrial septal integrity (size, morphology, continuity), shunt blood flow, major vascular dimensions, and valve motion. All diagnoses were jointly verified by two certified prenatal physicians after independent examinations. The ultrasound screenings were conducted during two critical periods: early pregnancy (11–13^+6^ weeks) for nuchal translucency (NT) measurement and chromosomal risk stratification, and mid-gestation (20–24 weeks) for detailed anatomical surveys, with a focus on detecting structural anomalies.

### 2.3 Karyotype analysis

Under the guidance of ultrasound localization, amniocentesis was performed through the abdominal wall. The specific steps are as follows: (1) Under the guidance of B-mode ultrasound, the position of the fetus in the uterus and the fetal heart was determined, and the optimal site for amniocentesis was selected. (2) After determining the site, local infiltration anesthesia was performed, and the amniocentesis needle was fixed under the guidance of B-mode ultrasound. (3) 10–20 ml of amniotic fluid was aspirated from the amniotic cavity and boiled in a water bath at 37 ℃. (4) The obtained amniotic fluid was centrifuged at 1,500 r/min for 6 min, and the supernatant was discarded under sterile conditions. The precipitate (0.5 ml) was mixed with 2 ml of amniotic fluid culture medium (BIO-AMF-2, Israel and domestic) and thoroughly mixed. (5) The mixture was inoculated into a 25 cm open cell culture bottle (BD Company) and cultured in a 5% CO₂, humidity-saturated incubator at 37 ℃. (6) After 7 days of static culture, the culture medium was replaced, and after another 24 h, the growth of amniotic fluid cells was observed. Cell collection, hypotonic treatment, fixation, slide preparation, and G-banding were performed. Karyotype analysis was conducted according to the International System for Human Cytogenetic Nomenclature (ISCN 2024) standards.

### 2.4 Chromosomal microarray analysis

Genomic DNA extracted from amniotic fluid samples was analyzed using the Agilent SurePrint G3 Human CGH + SNP 8 × 60 K microarray platform (Agilent Technologies, Santa Clara, CA) with the Agilent aCGH DNA Labeling Kit, achieving a resolution of 50 kb for deletions and 100 kb for duplications. Microarray hybridization and scanning were performed on an Agilent SureScan Dx scanner (3 μm resolution), and data analysis was conducted using Agilent CytoGenomics software (v5.1.2) with the ADM-2 algorithm (threshold = 6.0). All identified CNVs ≥200 kb (or smaller variants in clinically significant regions such as *22q11.21*) were validated by quantitative real-time PCR (qPCR) using TaqMan assays. The final CNV interpretations were issued by board-certified prenatal diagnosticians, with integrated analysis of multiple databases including DECIPHER, ClinVar, DGV, OMIM, and PubMed. CNV classifications followed ACMG 2020 guidelines, ISCN standards, and clinical expert consensus, with explicit notation of potential interpretation variations due to database updates.

### 2.5 Follow-up

All cases were followed up by telephone to understand the pregnancy outcomes and the prognosis of live-born infants. Low-risk pregnant women were followed up for 3–6 months after pregnancy, while high-risk pregnant women, especially those with clinically significant CNVs detected, were followed up for as long as possible.

### 2.6 Data processing and statistical analysis

Statistical analysis was performed using SPSS 25.0 software. Count data were expressed as number of cases (percentage). The comparison of chromosomal karyotype abnormality rates among groups was conducted using the *χ*^2^ test (Fisher's exact test). The comparison of VUS detection rates among groups was performed using Fisher's exact test, with the calculation of 95% confidence intervals (Clopper-Pearson exact method). The comparison of three groups of heart malformations (simple, complex, combined with extracardiac abnormalities) with the control group was corrected for multiple testing using the Bonferroni method. All statistical tests were two-sided, with *P* < 0.05 considered statistically significant.

## 3 Results

### 3.1 Heart malformations classification and ultrasound findings

In our study, 3,386 pregnant women underwent fetal heart examinations using ultrasound imaging, and 697 cases of heart malformations were identified. These cases were classified into nine categories based on the type of cardiac defect, as detailed in Table 1. The most common type was septal defects, which included ventricular and atrial septal defects, accounting for 44.48% of the cases (*n* = 310). Isolated valve abnormalities, such as mitral, tricuspid, aortic, and pulmonary valve regurgitation, were the second most common, comprising 40.60% of the cases (*n* = 283). Right ventricular outflow tract abnormalities, including tricuspid stenosis, tricuspid atresia, and pulmonary valve atresia, accounted for 3.16% (*n* = 22). Left ventricular outflow tract abnormalities, such as mitral valve stenosis, aortic valve stenosis, and interrupted aortic arch, constituted 1.72% (*n* = 12). Isolated conus arteriosus abnormalities, including Tetralogy of Fallot and complete transposition of the great arteries, were rare, accounting for 0.29% (*n* = 2). Complex conus arteriosus abnormalities, which included phenotypes from Group E with additional intracardiac anomalies, accounted for 0.86% (*n* = 6). Left ventricular hypoplasia syndrome was identified in 7.89% of the cases (*n* = 55). Situs anomalies syndrome was found in 0.43% (*n* = 3), and other anomalies, such as complete pulmonary venous return anomaly, single ventricle, single atrium, and persistent left superior vena cava, accounted for 0.57% (*n* = 4).

**Table 1:** Classification of heart malformations and ultrasound diagnosis results.

No.	Heart malformations type	Ultrasound diagnosis results	Case *n* (%)
A	Septal Defects	Ventricular Septal Defect, Atrial Septal Defect	310 (44.48)
B	Isolated Valve Abnormalities	Mitral Valve Regurgitation, Tricuspid Valve Regurgitation, Aortic Valve Regurgitation, Pulmonary Valve Regurgitation	283 (40.60)
C	Right Ventricular Outflow Tract Abnormalities	Tricuspid Stenosis, Tricuspid Atresia, Pulmonary Valve Atresia, etc.	22 (3.16)
D	Left Ventricular Outflow Tract Abnormalities	Mitral Valve Stenosis, Aortic Valve Stenosis, Interrupted Aortic Arch, etc.	12 (1.72)
E	Isolated Conus Arteriosus Abnormalities	Tetralogy of Fallot, Complete Transposition of the Great Arteries, Persistent Arterial Duct	2 (0.29)
F	Complex Conus Arteriosus Abnormalities	Phenotype from Group E with Additional Intracardiac Anomalies	6 (0.86)
G	Left Ventricular Hypoplasia Syndrome	Left Ventricular Hypoplasia Syndrome	55 (7.89)
H	Situs Anomalies Syndrome	Situs Anomalies Syndrome	3 (0.43)
I	Others:	Complete Pulmonary Venous Return Anomaly, Single Ventricle, Single Atrium, Persistent Left Superior Vena Cava, etc.	4 (0.57)

In accordance with the diagnostic criteria for birth defects established by the Ministry of Health of China and considering the severity of heart malformations, the 697 cases were categorized into four groups: simple heart malformations (602 cases, 86.37%), complex heart malformations (69 cases, 9.9%), heart malformations combined with structural extracardiac malformations (18 cases, 2.58%), and heart malformations combined with soft markers (8 cases, 1.15%). In our analysis, simple and complex heart malformations were treated as independent types.

### 3.2 Karyotype analysis results

Among 697 fetuses with prenatally diagnosed cardiac malformations, 66 lacked karyotype data and two cultures failed, leaving 629 evaluable cases; chromosomal abnormalities were detected in 41 (6.52%). These comprised 14 (2.23%) numerical aberrations—two trisomy 21, seven trisomy 18, two sex-chromosome aneuploidies, and three mosaics—and 27 (4.29%) structural anomalies, including deletions, duplications, inversions, translocations, and insertions. Under the revised classification, chromosomal abnormality rates increased stepwise: 4.74% (26/549) in simple malformations, 16.36% (9/55) in complex malformations, 27.78% (5/18) in those with structural extracardiac anomalies, and 14.29% (1/7) in those with soft markers; each was significantly higher than the control cohort (4.71%, *P* < 0.05), as shown in Table 2.

**Table 2:** Comparison of karyotype results between experimental and control groups.

Group	Normal Karyotype	Abnormal Karyotype	Culture Failure	Total	Abnormal Karyotype Positive Rate	*P*-value
Simple heart malformations	523	26	1	550	4.73%	0.032
Complex heart malformations	46	9	0	55	16.36%	0.014
Heart malformations combined with structural extracardiac malformations	13	4	1	18	22.22%	0.01
Heart malformations combined with soft markers	6	2	0	8	25.00%	0.05
Control Group	2,100	104	4	2,208	4.71%	-

### 3.3 Comparison of VUS and CNV detection rates

Among 697 fetuses with cardiac malformations, 644 underwent chromosomal microarray analysis. Pathogenic CNVs were identified in 34 cases (5.28%) and VUS in 9 (1.40%).Simple malformations (*n* = 553): CNV 4.88% (*P* < 0.05 vs. controls), VUS 0.54% (95% CI 0.04–1.29).Complex malformations (*n* = 65): CNV 7.69% (*P* = 0.36), VUS 1.54% (95% CI 0.38–10.70).Cardiac malformations with structural extracardiac anomalies (*n* = 24): CNV 8.33% (*P* = 0.033), VUS 12.50% (95% CI 6.55–39.35).Cardiac malformations with soft markers (*n* = 2): no CNV, VUS 100% (95% CI 29.2–100).Except for the soft-marker subgroup, all cardiac subgroups exhibited significantly higher CNV rates than the control cohort (1.38%). Cardiac malformations with structural extracardiac anomalies showed the highest CNV burden, and both this group and the soft-marker group displayed elevated VUS rates compared with other subgroups and controls. Overall, fetuses with cardiac malformations accompanied by structural extracardiac anomalies carry the greatest genetic risk, as shown in Table 3.

**Table 3:** Comparison of VUS and CNVs analysis between experimental and control groups.

Group	VUS	Total	VUS Detection Rate	95% CI	*P*-value^a^	Pathogenic CNV Detection Rate (*n*)	*P*-value^b^
Simple heart malformations	3	553	0.54%	0.04–1.29	-	4.88%(27)	<0.05
Complex heart malformations	1	65	1.54%	0.38–10.70	0.36	7.69%(5)	0.002
Heart malformations combined with structural extracardiac malformations	3	24	12.50%	6.55–39.35	<0.001	8.70%(2)	0.033
Heart malformations combined with soft markers	2	2	100%	29.2–100	-	0.00%(0)	-
Control Group	6	2,177	0.28%	0.10–0.60	-	1.38%(30)	-

^a^ *P*-value for comparison of VUS detection rates (Bonferroni-corrected).

^b^ *P*-value for comparison of pathogenic CNV detection rates (vs. control group, Fisher's exact test).

### 3.4 Genomic features and clinical manifestations of heart malformations fetuses with VUS

Among the 9 fetuses with heart malformations identified with VUS through chromosomal microarray analysis (CMA), the VUS segments ranged in size from 0.37 Mb–26.0 Mb, with significant variations in gene content and clinical manifestations across different groups. In the complex heart malformations group, Case 9 exhibited a large 21.2 Mb deletion on *2q31.1q32.3*, associated with Tetralogy of Fallot, suggesting a high gene load and potential contribution to the complex phenotype. The simple heart malformations group (Cases 2, 6, and 8) had smaller VUS segments ranging from 0.37 Mb–1.07 Mb, associated with relatively mild clinical features such as mild tricuspid regurgitation and small ventricular septal defects. The heart malformations with structural extracardiac anomalies group (Cases 4, 5, and 7) had larger VUS segments ranging from 2.13 Mb–26.0 Mb, associated with more severe clinical manifestations including bilateral lateral ventricular widening, fetal intestinal dilation, and clubfoot. The heart malformations with soft markers group (Cases 1 and 3) had VUS segments of 2.47 Mb and 2.55 Mb, respectively, associated with soft markers such as fetal nasal bone absence and intrahepatic punctate strong echo. Statistical analysis revealed that the median size of VUS segments in the structural extracardiac anomalies group was 2.28 Mb (IQR 2.13–26.0), significantly larger than the 0.49 Mb (IQR 0.37–1.07) in the simple heart malformations group (Mann–Whitney U test, *P* = 0.028). The complex heart malformations group had the largest segment (21.2 Mb). Gene load analysis showed that the structural extracardiac anomalies group and the complex heart malformations group had higher average gene loads (5.2 ± 3.1) compared to the simple heart malformations group (1.5 ± 0.7) (*t*-test, *P* = 0.02). The soft markers group had an intermediate gene load (2.5 ± 0.7). These findings suggest a significant association between larger VUS segments and more severe clinical phenotypes, with the structural extracardiac anomalies and complex heart malformations groups exhibiting larger VUS segments and higher gene loads, potentially contributing to their complex clinical presentations. Further functional studies and clinical validation are required to confirm the pathogenicity of these VUS segments and their impact on clinical outcomes, as shown in Table 4.

**Table 4:** VUS analysis of 9 cases of heart malformations in fetuses.

Case	Age (years)	Gestational Age (weeks)	Ultrasound Findings	CMA Result	VUS	Length (Mb)	Suspected Pathogenic Genes
1	25	24 + 4	Fetal nasal bone absent, tricuspid regurgitation	Normal	14q22.1q22.2 Heterozygous Deletion	2.47 Mb	*BMP4, PTX2, SIX6, GCH1, TMEM260*
2	31	24 + 3	Mild tricuspid regurgitation, left ventricular focal strong echo?	Normal	17q11.2 Duplication	0.49 Mb	*NF1*
3	38	23 + 2	Fetal intrahepatic punctate strong echo, possible calcification, tricuspid regurgitation, strong echo in both ventricles	Normal	2p16.1p15 Duplication	2.55 Mb	*BCL11A, PAPOLG, REL*
4	28	24 + 5	Bilateral lateral ventricular widening, tricuspid regurgitation	Normal	12q15q22 Heterozygous Deletion	26Mb	*OTOGL, BBS10, ALX1*
5	27	30 + 5	Fetal intestinal dilation, bilateral renal pelvis separation, persistent left superior vena cava, tricuspid regurgitation, left ventricular focal strong echo	Not Done	18q23 Heterozygous Deletion	2.13 Mb	*TXNL4A*
6	32	24 + 2	Fetal ventricular septal defect, tricuspid regurgitation, persistent left superior vena cava	Not Done	22q11.21 Duplication	0.37Mb	*HNF1B*
7	28	24 + 5	One twin of a dichorionic diamniotic pregnancy with absent venous duct, clubfoot, single umbilical artery, persistent left superior vena cava	Normal	5q22.1q22.2 Duplication	2.28 Mb	-
8	27	26 + 3	Fetal ventricular septal defect 2mm	Normal	13q12.2q12.3 Duplication	1.07 Mb	*PAN3, FLT1, POMP, SLC46A3*
9	29	18 + 6	One twin of a pregnancy with Tetralogy of Fallot (VSD, overriding aorta, pulmonary stenosis)	Normal	2q31.1q32.3 Heterozygous Deletion	21.2 Mb	

### 3.5 Pregnancy outcome follow-up

All 697 pregnant women were followed up by telephone. The follow-up results showed that 612 cases (87.80%) chose to continue the pregnancy and delivered live-born infants, 61 cases opted for elective abortion, 3 cases had stillbirth abortions, and 21 cases refused follow-up or had incorrect contact information. Among the 61 aborted fetuses, the majority were fetuses with genetic syndromic CHD and complex CHD. Among the 612 live-born infants, 9 cases were found to have extracardiac structural abnormalities, including hypospadias and microcephaly, polydactyly, cleft lip and palate, mandibular hypoplasia, external ear deformities, and unilateral renal agenesis. One case died postnatally due to metabolic abnormalities. The remaining newborns were generally in good condition, with no neurological developmental disorders or other abnormalities detected. Follow-up at 3–6 months postnatally revealed no other significant abnormalities.

Among the 9 cases carrying VUS: 6 cases chose to continue the pregnancy, including 2 cases of isolated heart malformations and 4 cases of heart malformations combined with structural extracardiac malformations, and no abnormalities were found in the live-born infants during follow-up. In the 3 cases that chose elective abortion, 1 case was due to multiple system malformations, and 2 cases were non-isolated cardiac abnormalities. Among the three pregnancies terminated due to VUS, parental interviews revealed the following: (1), Case 1 (14q22.1q22.2 heterozygous deletion): Termination was chosen due to anxiety about uncertain neurodevelopmental risks, despite genetic counseling; (2) ,Case 5 (18q23 heterozygous deletion):The decision was driven by combined heart malformations, intestinal dilation, and renal anomalies, with VUS serving as a contributing factor; (3), Case 9 (2q31.1q32.3 heterozygous deletion): Termination was primarily attributed to the severity of tetralogy of Fallot (TOF), with VUS amplifying parental concerns.

## 4 Discussion

### 4.1 Distribution of VUS in different types of heart malformations

Recent studies have underscored the utility of CMA in detecting CNVs in fetuses with cardiovascular malformations (CVM). A systematic review and meta-analysis of CMA in fetuses with isolated CVM reported an overall diagnostic yield of 5.79% (CI 5.54–6.04) (10). Our study observed a diagnostic yield of pathogenic CNVs of 5.30% (34/644), which is comparable to the aforementioned meta-analysis. The meta-analysis also provided detailed diagnostic yields for various CVM subgroups: conotruncal defects (CTD) had the highest detection rate at 15.93% (CI 15.75–16.11), followed by tetralogy of Fallot (TOF) at 11.28% (CI 9.7–12.86), left ventricular outflow tract defects (LVOTD) at 6.67% (CI 5.51–7.83), hypoplastic left heart syndrome (HLHS) at 6.52% (CI 5.64–7.40), dextro-transposition of the great arteries (D-TGA) at 6.49% (CI 5.26–7.72), right aortic arch (RAA) at 4.42% (CI 2.36–6.48), ventricular septal defects (VSD) at 2.64% (CI 2.26–3.02), and aberrant right subclavian artery (ARSA) at 0.66% (CI 0.62–0.70). These findings highlight the significance of CMA in identifying genetic anomalies in specific types of heart malformations, particularly those with higher detection rates such as CTD and TOF.

CMA serves as the first-line tool for prenatal genetic diagnosis of heart malformations, with indications including cardiac structural abnormalities and multisystem malformations. By detecting CNVs, it significantly increases the detection rate of genetic etiology in fetal heart malformations, especially in non-isolated heart malformations (combined with extracardiac abnormalities). The diagnostic rate varies from 4%–28% in different studies, with some subtypes (such as atrioventricular septal defects and conotruncal anomalies) reaching up to 27%–28% (11). Compared with karyotype analysis, CMA can additionally detect 5%–11.9% of submicroscopic CNVs (such as microdeletions/microduplications), compensating for the resolution limitations of traditional karyotyping (12). A study screening 7,150 pregnant women samples reported a VUS detection rate of 1.05% (75/7,150) (13). In our study, 2,821 cases underwent CMA testing, with a total of 15 VUS identified, accounting for only 0.53%. Among the 644 fetuses with heart malformations, the proportion was 1.40%. These differences suggest that the detection rate of VUS may be influenced by sample characteristics and testing conditions. Therefore, in clinical practice, the interpretation of VUS should be integrated with specific clinical contexts and multidisciplinary team opinions to provide more accurate genetic counseling.

VUS are less common in isolated heart malformations but more frequently seen in non-isolated heart malformations or fetuses with other ultrasound abnormalities (such as multisystem malformations) (14). It has been reported that some VUS may be associated with specific heart malformations subtypes (such as ventricular septal defects and conotruncal anomalies), but further functional validation is needed (15). In our study, we identified two VUS related to complex heart malformations, whose phenotypic characteristics were highly similar to previous reports. One fetus presented with ventricular septal defect (VSD), accompanied by tricuspid regurgitation and persistent left superior vena cava; the other was diagnosed with tetralogy of Fallot (TOF), a typical conotruncal anomaly.

Our study demonstrates that the distribution of VUS varies significantly among different types of heart malformations. The detection rate of VUS was lowest in simple heart malformations (0.54%, 3/553), followed by complex heart malformations (1.54%, 1/65), and highest in heart malformations combined with structural extracardiac malformations (12.50%, 3/24), while heart malformations combined with soft markers showed a VUS rate of 100% (2/2). This suggests that different types of heart malformations may involve different genetic backgrounds. Simple heart malformations has relatively lower genetic heterogeneity, while complex heart malformations and heart malformations combined with structural extracardiac malformations involve more complex genetic factors and a greater number of genetic variations. This is consistent with previous studies, which concluded that in non-isolated heart malformations and fetuses with ventricular septal defects, CMA is more likely to detect submicroscopic structural abnormalities (including VUS), indicating a more complex genetic background in these types of heart malformations and the potential involvement of more unknown pathogenic variations (16). Another study also confirmed that individuals carrying CNVs all had heart malformations combined with other major malformations, and 23.5% of cases detected VUS, indicating that heart malformations combined with other abnormalities is more likely to be associated with genetic variations (including VUS) (17). The detection rate of single-gene variations in complex heart malformations is significantly higher than that in simple heart malformations (36.4% vs. lower detection rate), and the overall diagnostic rate is higher in cases with multisystem involvement (OR = 2.41), further supporting the association of complex heart malformations with a higher frequency of genetic abnormalities (18). The genetic contribution to simple heart malformations mainly comes from the cumulative effect of common variations, while the detection rate of rare variations (such as VUS) is relatively low, consistent with the lowest VUS detection rate in simple heart malformations in our study (19).

We also found that in patients with complex heart malformations and heart malformations combined with structural extracardiac malformations, the positive rate of karyotype abnormalities and the detection rate of pathogenic CNVs were significantly higher than those in patients with simple heart malformations and fetuses without heart malformations. These two types of genetic variations often involve large segmental deletions or duplications, indicating that simple fetal heart malformations may have a different pathogenic mechanism from the other two types. This may be because these cases contain more gene regions related to known syndromes, and variations in these regions are more likely to be detected as CNVs and VUS, thereby increasing the complexity of genetic counseling. Previous studies have shown that VUS related to complex heart malformations are often concentrated in regions such as *3q29, 5q22.1-22.2, and 9p22*, which may have potential associations with gene regulatory networks related to cardiac development (20). In our study, one VUS related to complex heart malformations was manifested as a *2q31.1q32.3* heterozygous deletion, while the remaining eight VUS related to simple heart malformations or non-isolated heart malformations involved microdeletions or microduplications on chromosomes 2, 5, 12, 13, 14, 17, and 18, with lengths ranging from 0.37 Mb–26 Mb. Through comparison and analysis with multiple databases, we found that among the nine VUS, three could be retrieved with gene variations related to the occurrence of fetal heart malformations (such as *NF1, HNF1B, MAP3K20*), and five VUS contained multiple protein-coding genes, which are mostly associated with diseases such as special facial features, oral and lip malformations, reproductive system malformations, and intellectual disabilities (21–24). However, one VUS still could not detect the expression of related genes in the existing gene databases. These findings provide new data support for genetic counseling, but their pathogenicity still needs to be confirmed through further research. In summary, VUS in different types of heart malformations show different characteristics in terms of gene region size and association with known syndromes. VUS in simple heart malformations mostly involve smaller gene regions, while VUS in complex heart malformations and heart malformations combined with structural extracardiac malformations involve larger gene regions, and the latter are mostly related to known syndromes.

### 4.2 Impact of VUS on clinical decision-making

The uncertainty associated with VUS may prompt parents and healthcare teams to choose to terminate the pregnancy based on potential risks, despite insufficient evidence, primarily due to psychological stress, misinterpretation, and the lack of standardized guidelines. A qualitative study showed that even when the objective pathogenic risk of VUS is low, its ambiguity can still lead to a persistent psychological burden for parents, with some choosing to terminate the pregnancy due to an inability to tolerate this uncertainty (25). Although all women ultimately felt satisfied with their decisions, the uncertain information itself can trigger intense psychological conflicts. Another study involving 94 fetuses showed that 14.9% (14 cases) carried VUS; among these, 88 pregnancies were terminated, with only 6 continuing (26). Moreover, the consultation approach may also affect decision-making, with some physicians tending to provide “all-options counseling” (explicitly discussing the options to continue or terminate the pregnancy), while others adopt “choice-based counseling” (prioritizing the assessment of patient preferences) (27). These studies highlight the direct association between VUS and the decision to terminate the pregnancy: many parents and healthcare teams tend to choose termination after a VUS diagnosis to reduce potential health risks, even when the pathogenicity of VUS is not yet clear. However, a clinical study that followed up on 139 cases of VUS found that only 6.5% were subsequently reclassified as pathogenic, and some of the children who were born did not exhibit the expected phenotypes (28). This suggests that the phenotypic expression rate of fetuses carrying VUS may be overestimated, and decisions to terminate the pregnancy based on this may involve the risk of over-intervention.

Our study indicates that simple heart malformations has a favorable prognosis and a high live birth rate, providing strong support for families to choose to continue the pregnancy. Research has shown that simple heart malformations is usually associated with a lower risk of genetic abnormalities and a better prognosis, while complex heart malformations or cases combined with extracardiac abnormalities have a poorer prognosis, which may be related to the lower detection rates of CNVs and VUS in simple heart malformations (29). Another study also demonstrated that the detection rate of genetic abnormalities in simple heart malformations in postnatal screening is low and not directly related to VUS, suggesting a relatively simple genetic background (30). Moreover, the neonatal outcomes of pregnant women with simple heart malformations are similar to those of the general population, and the recurrence risk for offspring is low (1.9%), which may be related to the lighter genetic burden of simple heart malformations (31). In contrast, complex heart malformations and heart malformations combined with structural extracardiac malformations have higher rates of elective abortion, and their management requires multidisciplinary risk assessment in combination with CMA results (such as pathogenic CNVs and VUS). Existing studies have confirmed that the use of CMA or exome sequencing (ES) technology can significantly increase the detection rate of VUS, especially when traditional karyotype analysis results are normal (32, 33). A meta-analysis pointed out that the detection rate of VUS in heart malformations is about 4%, and it is higher in non-isolated or complex heart malformations (16). However, the detection rate of VUS in heart malformations in this study is about 1.5%, and this lower detection rate may be related to the fact that some cases did not undergo CMA analysis.

While our study found an association between larger VUS segments and more severe clinical phenotypes, it is important to note that this association does not imply causation. Functional validation through additional experiments, such as cell line studies or animal models, is needed to confirm the pathogenicity of these VUS segments. In complex heart malformations, parents are more likely to choose to terminate the pregnancy. In heart malformations combined with structural extracardiac malformations carrying VUS, due to the possible poor prognosis, parents are often anxious and confused when facing uncertain results, and more genetic counseling and multidisciplinary team opinions are needed to make decisions.

Complex heart malformations is often associated with chromosomal abnormalities or CNVs. Taking complex heart malformations such as tetralogy of Fallot as an example, the study of its molecular mechanism needs to be combined with genetic syndrome or non-syndrome background, which suggests that VUS may play an important role in the etiology of complex heart malformations (34). In fact, current research reports show that the mortality rate of patients with complex heart malformations is positively correlated with the complexity of the disease, and this finding indirectly supports the impact of genetic background (such as VUS) on prognosis (35). In addition, the risk of postoperative complications for patients with complex heart malformations is 2.1 times that of non-heart malformations patients, which may be related to the anatomical or physiological complexity caused by potential genetic abnormalities VUS (36). Therefore, in complex heart malformations, the clinical significance of VUS usually needs to be determined in combination with additional phenotypic or family history analysis. In particular, for fetuses with complex heart malformations carrying VUS, if combined with extracardiac abnormalities, their pathogenic risk needs to be assessed with emphasis (37). In contrast, the interpretation of VUS in isolated heart malformations relies more on functional studies or population frequency data (38).

Non-isolated heart malformations has a stronger association with chromosomal abnormalities, and its exome sequencing (ES) diagnostic rate is significantly higher than that of isolated heart malformations (14.7% vs. 11.5%), indicating that VUS or other submicroscopic variations are more likely to occur in such cases (29). Another study also pointed out that the genetic basis of non-isolated heart malformations is more likely to involve polygenic or epigenetic factors, rather than simple or isolated types (30). Therefore, it is recommended to conduct prenatal and postnatal genetic counseling for patients with non-isolated heart malformations to identify VUS and assess its clinical significance, especially in cases combined with other malformations (39).

At present, CMA has become the core tool for prenatal genetic diagnosis of heart malformations, but the accompanying issue of VUS highlights the technical limitations and clinical complexity. The current dilemma in clinical decision-making mainly stems from the uncertainty of the pathogenicity of VUS, especially in heart malformations combined with structural extracardiac malformations, where the high detection rate of VUS further increases the difficulty of decision-making, posing a great challenge to genetic counseling. At the same time, there is still controversy over whether to routinely perform CMA detection for all heart malformations fetuses. Some studies suggest stratified detection based on heart malformations subtypes, prioritizing non-isolated heart malformations fetuses, as the detection rate of VUS in isolated heart malformations fetuses may be higher than that of CNVs (40). In addition, different laboratories have different classification criteria for VUS, which may affect the interpretation and clinical application of the results (41). In simple heart malformations, although the detection rate of VUS is low, most families choose to continue the pregnancy due to the relatively good prognosis, and the phenotypes of these fetuses carrying VUS are mostly normal after birth (42). However, there are also cases where pathogenic variants are missed, leading to delayed intervention (43). In complex heart malformations, parents are more likely to choose to terminate the pregnancy. In heart malformations combined with structural extracardiac malformations carrying VUS, due to the possible poor prognosis, parents are often anxious and confused when facing uncertain results, and more genetic counseling and multidisciplinary team opinions are needed to make decisions.

In response to the current dilemma in managing VUS, it is recommended to combine fetal phenotypes (such as types of cardiac defects, whether combined with other abnormalities) and family history to assess the potential risks of VUS in prenatal management (11). For cases of isolated heart malformations carrying VUS, the indications for CMA should be strictly limited to avoid uncertainty caused by over-testing (13). In terms of technical detection, whole exome sequencing (WES) can be used to assist in interpreting the clinical significance of VUS, especially in cases where CMA is negative but the phenotype is complex (39), and functional experiments (such as animal models or cell line validation) are also key steps in confirming the pathogenicity of VUS (40). The focus of genetic counseling is to clearly explain the “uncertainty” of VUS to families and discuss the recurrence risks of subsequent pregnancies and testing options (such as targeted CNV detection or family co-segregation analysis) (41). In the face of insufficient long-term follow-up data for VUS, standardized databases can be established to improve classification (42). Future research directions include developing heart malformations-specific CMA chips to integrate known pathogenic CNV regions and VUS hotspots, as well as exploring phenotype-genotype associations and combining multi-omics data (such as metabolomics, epigenetics) to enhance the interpretation of VUS (43). At the same time, it is necessary to optimize the testing process through multidisciplinary collaboration (radiology, genetics, bioinformatics) and establish standardized management guidelines for VUS to balance diagnostic benefits with potential risks.

In summary, the clinical interpretation of VUS in fetal heart malformations needs to combine fetal phenotypes (types of cardiac malformations, whether combined with extracardiac abnormalities), genomic features (such as segment size, gene load), and family genetic data to avoid overdiagnosis or missed diagnoses. Simple heart malformations has fewer genetic abnormalities, weaker VUS associations, high live birth rates, and a good prognosis; in complex heart malformations and cases combined with extracardiac abnormalities, the detection rates of VUS or CNVs are significantly increased (3.08% for complex type and 19.23% for combined abnormality group), and it is necessary to clarify the genetic background through CMA or whole exome sequencing (WES) and jointly assess the prognosis and intervention plans with multidisciplinary teams including radiology, genetics, pediatrics, and obstetrics.

## 5 Limitations and future perspectives

The limitations of this study lie in the relatively small sample size and the short follow-up duration. Future research directions include: Increasing the sample size: A larger sample size would allow for a more in-depth investigation into the distribution patterns of VUS in different types of heart malformations, thereby enhancing the statistical significance of the results. Combining parental validation and functional experiments: By conducting parental validation and functional experiments, the pathogenicity of VUS can be clarified, thereby reducing the impact of uncertain results on clinical decision-making. Improving CNV databases: Establishing more comprehensive CNV databases would provide a more accurate basis for clinical decision-making.

## 6 Conclusion

Fetuses with cardiac malformations accompanied by structural extracardiac anomalies carry the highest genetic risk; karyotyping combined with CMA should therefore be performed routinely. Complex cardiac malformations also warrant concurrent testing, whereas simple malformations and those with soft markers can be evaluated individually.

## Data Availability

The raw data supporting the conclusions of this article will be made available by the authors, without undue reservation.
